# A Mechanochemical Model for Embryonic Pattern Formation: Coupling Tissue Mechanics and Morphogen Expression

**DOI:** 10.1371/journal.pone.0082617

**Published:** 2013-12-20

**Authors:** Moritz Mercker, Dirk Hartmann, Anna Marciniak-Czochra

**Affiliations:** 1 Institute of Applied Mathematics, BioQuant and Interdisciplinary Center of Scientific Computing (IWR), University of Heidelberg, Heidelberg, Germany; 2 Interdisciplinary Center of Scientific Computing (IWR), University of Heidelberg, Heidelberg, Germany; 3 Siemens AG, Corporate Technology, Munich, Germany; University of Cambridge, United Kingdom

## Abstract

Motivated by recent experimental findings, we propose a novel mechanism of embryonic pattern formation based on coupling of tissue curvature with diffusive signaling by a chemical factor. We derive a new mathematical model using energy minimization approach and show that the model generates a variety of morphogen and curvature patterns agreeing with experimentally observed structures. The mechanism proposed transcends the classical Turing concept which requires interactions between two morphogens with a significantly different diffusivity. Our studies show how biomechanical forces may replace the elusive long-range inhibitor and lead to formation of stable spatially heterogeneous structures without existence of chemical prepatterns. We propose new experimental approaches to decisively test our central hypothesis that tissue curvature and morphogen expression are coupled in a positive feedback loop.

## Introduction

During the development of biological tissues, different signaling molecules are responsible and indispensable for pattern formation and shape generation [1]. Since the seminal paper of Alan Turing [2] a variety of patterns in biological tissues have been studied using a framework of reaction-diffusion equations. These approaches assume that there exist diffusing signaling molecules, called morphogens, whose nonlinear interactions combined with different rates of diffusion lead to destabilization of a spatially homogeneous equilibrium and formation of spatially heterogeneous structures.

The idea looks counter-intuitive, since diffusion is expected to lead to a uniform distribution of molecules. Mathematical analysis of reaction-diffusion equations provides explanation of the phenomenon postulated by Turing. Patterns arise through a bifurcation, called diffusion-driven instability, in which a spatially homogeneous stationary solution looses stability for a certain range of diffusion coefficients and a stable spatially heterogenous stationary solution appear. The resulting structures can be monotone corresponding to the gradients in positional information or spatially periodic and their ultimate shape depends on the diffusive scaling, related to the size of domain.

The most famous embodiment of Turing's idea in a mathematical model of biological pattern formation is the activator-inhibitor model proposed by Gierer and Meinhardt [3]. One of the key ingredients of that model, responsible for Turing type dynamics, is that the inhibitor diffuses faster than the activator, i.e. the system is regulated by a short range activation and a long range inhibition [2], [3]. However, in many developmental processes, dynamic and complex tissue topologies are likely to prevent the establishment of long range inhibitor gradients [4]. Furthermore, diffusion coefficients of typical morphogens are often found to be quite small [5], i.e. do not allow existence of significantly varying diffusion rates as required by the classical Turing mechanism. These observations suggest a search for a different inhibitory mechanism such as mechanical inhibition [4]. Moreover, mechanically based laws in morphogenesis appear to be promising and powerful alternatives to purely chemical models: The latter reduce macroscopic structures to ‘blind’ by-products of spatial chemical patterns and contradict certain experimental data [6], [7].

The influence of morphogens on tissue mechanics (such as curvature) is well known. However, different studies show that the interplay is reciprocal and mechanical stress and physical forces (e.g. induced by tissue deformations) can also locally influence morphogen patterns and cell behavior [4], [6], [8]–[12]. Furthermore, it appears that tissues may act differently depending on directions of applied stress [13].

Based on these observations, we propose a model for pattern formation in biological tissues, directly coupling the expression of a morphogen with tissue mechanics. Numerical simulations of the model based on our hypothesis reveal that

- simple interplay between tissue mechanics and morphogen production can lead spontaneously to curvature and morphogen patterns with shapes depending on the size of the tissue; and
- resulting patterns are insensitive to stochastic perturbations of initial conditions.

The proposed mechanism is a promising candidate to replace the “missing” long range inhibitor in the activator-inhibitor models. Due to its robustness, the ultimate shapes are reliably generated under various conditions. Therefore, the model can explain self-organization and *de novo* pattern formation in the systems with the initial state close to an equilibrium.

The mechanism is currently under experimental investigation. Furthermore, the presented mathematical model and numerical approach could be used in future work to investigate the interplay of different morphomechanical models such as those discussed in ref. [6].

**Figure 1:**
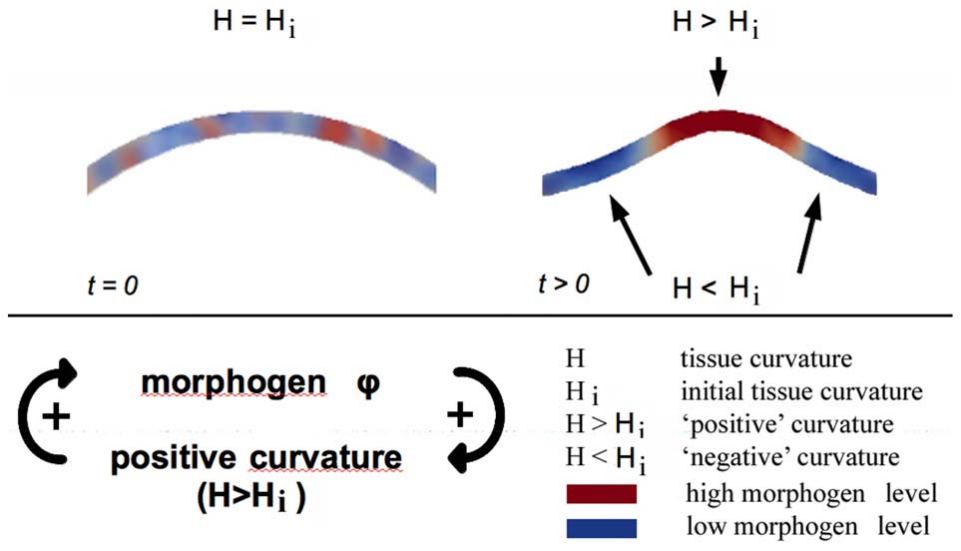
Positive feedback loop of morphogen expression and tissue curvature. In combination with morphogen degradation, this mechanism leads to spontaneous curvature and morphogen patterns starting from stochastic initial conditions. Note that positive curvature induces negative curvature at the edges of the domain, replacing the effect of a long range inhibitor molecule. Red and blue color depict high and low morphogen levels, respectively.

Our mathematical model combines a reaction-diffusion equation for the morphogen with an elastic gradient flow for tissue mechanics. Expanding the ideas of Cummings [14] we assume that the morphogen locally induces positive curvature, and in turn, this curvature induces the expression of this morphogen (c.f. Fig. 1), i.e. that there exists a positive feedback loop. Above, the terms “positive” and “negative” curvature refer to outward and inward bending, respectively, compared to the initial curvature of a tissue. More accurately, experimental data indicate that it is not tissue curvature itself but curvature modulated tissue compression that influences gene expression [4], [11]. However, in order to reduce the complexity of the model and to present the basic idea, we adhere in this paper to the simplified model. Models including tissue compressibility will be subject of future research.

Finite element simulations reveal that the postulated mechanism produces spontaneously a variety of curvature and morphogen patterns in an asymptotically stable way, i.e. insensitive to small changes in initial conditions. Based on a range of simulation results, we present detailed parameter studies of the model analyzing the rescaled parameter space. We identify parameters that control pattern-related scales, such as size, amount and curvature of appearing patches. Our results show that biomechanical interactions may constitute the missing link to the Turing long range inhibitor: positive curved domains induce negative curvatures at their edges; in these regions only morphogen degradation takes place (c.f. Fig. 1).

Furthermore, numerically obtained results show that dynamics as well as final curvature and morphogen patterns appear to be very similar to those observed during symmetry break in *Hydra* tissues. Thus, numerical results suggest that the presented mechanism could constitute a key mechanism for pattern formation in *Hydra* polyps. Further experimental work is needed to support the idea that the proposed mechanism indeed constitutes a key mechanism of pattern formation during embryogenesis. Corresponding experiments are suggested at the end of this paper indicated by simulations.

## Materials and Methods

### Model

Let 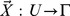 be a time-dependent parametric representation of a closed cell sheet 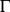, where the cell sheet is parameterized over the unit sphere adopting the ansatz of [15], , i.e. 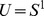. The morphogen level is described by the function 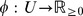; for each 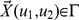 the concentration 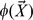 is identified with 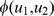. Hence, 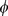 is naturally moving with the deforming tissue, i.e. 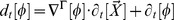, where 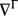 is the surface gradient. For convenience of the reader, detailed definitions of the used geometrical quantities are given in the Table S1 available online.

To model the curvature-dependent elastic properties of a thin cell tissue, we use a modified Helfrich energy, i.e.
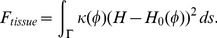
(1)Here, 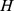 is the mean curvature and 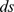 is the surface measure. 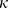 constitutes the bending rigidity reflecting the stiffness of the surface, and the spontaneous curvature 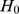 reflects the preferred local surface curvature. The latter e.g. depends on the shape of biological cells within the tissue: If cells are symmetrical, the tissue energetically favors a flat geometry and 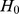 vanishes. However, in the case of wedge-shaped cells, the tissue prefers to be locally curved, hence 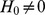 holds. A detailed treatment considering the connection between the geometry of biological cells arranged in a tissue to Helfrich measures such as 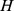 can be found in [17]. Since laterally heterogeneous distributed molecules -such as morphogens- may influence the mechanical properties of the tissue, we assume (analogously to [18], [19]) that the mechanical moduli 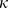 and 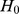 are functions of the concentration 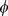. In first approximation we take 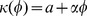 and 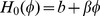 as linear functions of 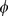, where 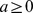. The original Helfrich energy 
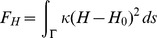
 [20] with constant moduli 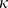 and 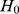 has been widely used describing the mechanics of membranes. In the following, we assume a local tissue incompressibility.

Adopting an energy point of view, the evolution of the tissue deformation 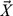 up to time 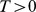 in 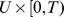 is given by the following 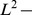
gradient flow under the constraint of local incompressibility of the tissue:
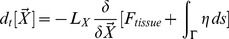
(2)

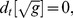
(3)where 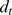 is the total time derivative, 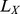 is a kinetic coefficient, 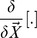 denotes the variation with respect to the arbitrary vector 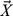, and 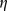 is a local Lagrange multiplier [21] due to the constraint of incompressibility (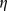 can be interpreted as pressure). Furthermore, 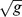 is the surface measure where 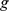 is the determinant of the first metric tensor (c.f. Table S1). Volume constraints are not considered (to account for the fact that in pattern formation experiments with *Hydra* tissues it appears that tissue spheres exchange internal fluid with their surroundings [22]). The gradient flow (2) leads to minimization of the free energy 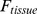 under the constraint of incompressibility (3). For detailed calculation of the variations of 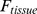 we refer to [19].

Let us now consider the dynamics of the morphogen 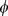 within the tissue. In contrast to the modeling of membrane dynamics showing fluid behavior with respect to lateral flows, the evolution of morphogens is modeled separately rather than obtained by a corresponding variation of free energy. Beside the basic assumptions concerning diffusion and degradation [5], [23], [24], we define the morphogen production as a function depending on the surface curvature. Furthermore, we assume that initially the tissue is arranged in a mechanically relaxed configuration with curvature 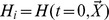. Experimental findings reveal that tissue deformations may induce morphogen production [4], [8], [9]. Thus, we postulate that the expression of 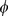 can be induced by local curvatures 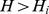. If both, negative and positive curvatures had induced morphogen expression, i.e. 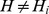, we would have expected oscillations instead of stable patterns. Hence, 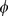 induces local positive curvatures via 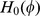 and positive curvatures induce locally the expression of 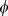 via appropriate reaction-terms, constituting a positive feedback loop (c.f. Fig. 1). Such positive feedback loops between chemical and mechanical processes during development have just been reported [12].

Using Michaelis-Menten kinetics (assuming the existence of a maximal expression rate of the 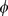-promoter) and defining 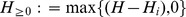, we obtain the dynamical equation for 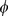:
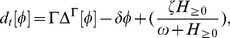
(4)


with constants 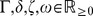. Hence, the model is given by a nonlinear PDE system of fourth order, coupling the gradient flow for tissue mechanics (2) under the constraint of local area incompressibility (3) with the reaction-diffusion equation (4) for morphogen dynamics.

### Nondimensionalization

In the following subsection the free energy (1) and corresponding dynamic equations (2)–(4) are nondimensionalized. This reformulation allows us, on the one hand, to identify essential parameters and characteristic properties of our system. On the other hand, it enables us to study the relative dependence of different parameters on spatial and temporal scales. To nondimensionalize the model, we replace each variable 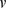 with a dimensionless quantity 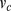 which is scaled with a characteristic unit of measure 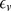.

*Free Energy*: We set 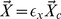 with 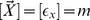 which implies 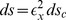, 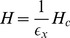, 
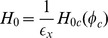
 with 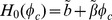 setting 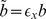 and 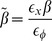 as well as 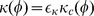 with 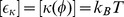. It follows:
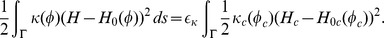



A consistency check shows that 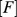 has units of energy as expected, i.e. 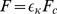 with 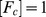. Calculating the variation of 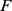 we obtain for the strong formulation with 
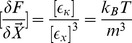
.

*Surface dynamics*: We set 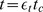 with 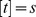. Thus it holds 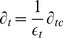 and 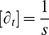. Choosing 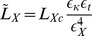 it follows
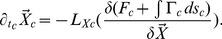
(5)


*Morphogen dynamics:* Choosing 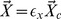 implies 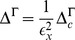 and 
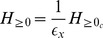
. Setting 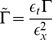, 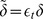, 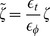, 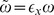 as well as 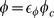 with 
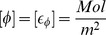
, we obtain
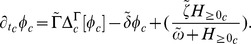



Choosing 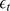 and 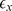 appropriately we can always guarantee 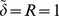. Furthermore, simulations show that equilibirum patterns are independent of 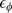 (c.f. Fig. 1 C–D). Hence, choosing 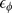 appropriately we can always yield 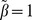. Moreover, restricting ourselves to the case 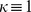 (i.e. 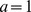 and 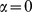) and 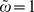 as well as choosing 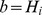 for biological reasons the total parameter space can be reduced to three independent constants, namely 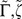 and 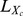.

### Finite element approximation and parameter setup

Using the outlined modeling approach, we investigate numerically the effect of different model parameters, system sizes and initial conditions on pattern formation. To do so, we approximate equations (2)–(4), and more accurately, the nondimensionalized form derived in the previous subsection, to study systematically spontaneous pattern formation induced by the proposed mechanism. The corresponding total parameter space can be reduced to three independent constants 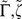 and 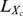.

For spatial discretization we use a biqubic mixed parametric finite element method. The tissue surface is discretized using a quadrangular grid with 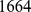 grid points. For time discretization we use an adaptive semi-implicit Euler scheme. For further details to the computation scheme we refer to ref. 16, 19.

Related to the early shape of an embryo (a blastula) we assume as the initial tissue geometry the sphere 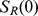 with 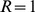, choosing an appropriate nondimensionalization, c.f. previous subsection. Moreover, we use a stochastic distribution for the initial morphogen concentration. The corresponding values at each discretization point are chosen using the standard random generator provided by C++. 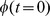 is uniformly distributed in an interval 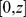 with the average 
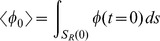
.

In the presented study, we restrict our studies to the case of morphogens influencing tissue curvature but not tissue rigidity, setting 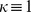 (i.e. 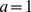 and 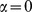). Moreover, we set 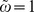, since neglecting long time behavior, i.e. nonlinear effects, it is sufficient to vary 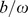 and 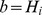. This latter represents the assumption that the tissue is initially arranged in a mechanically relaxed configuration. Furthermore, due to an appropriate nondimensionalization, we can always guarantee 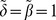 (c.f. previous subsection).

## Results and Discussion

**Figure 2:**
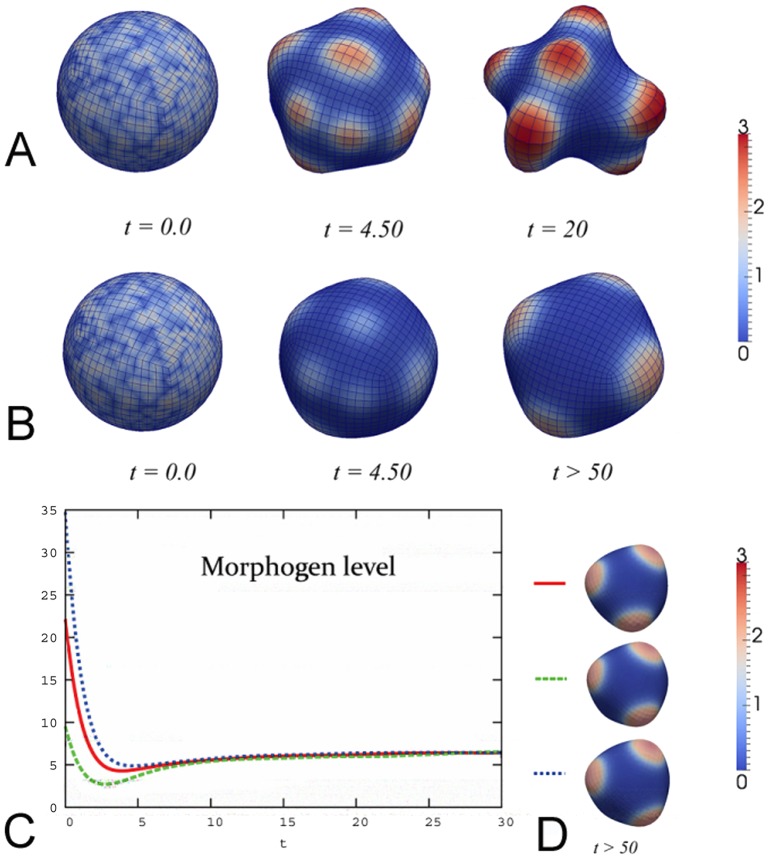
A–B: Simulations of spontaneous tissue pattern formation starting from stochastically distributed morphogen on a sphere at different time steps. A: For a strong coupling between curvature and morphogen expression the system shows strong budding (numerics do not perform properly for 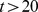 since the approach is only able to handle medium geometry changes; the likely equilibrium shape consists of fully budded morphogen patches). B: For a weak coupling between curvature and morphogen expression the system reaches a symmetric mechanical equilibrium at 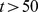. C–D: Equilibrium patterns are insensitive to different choices of initial conditions. C: Plot of total morphogen level 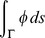 during tissue development for different values 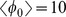 (green dashed line), 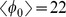 (red line) and 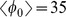 (blue dotted line). D: Corresponding equilibrium patterns. In all simulations red and blue color depicts high and low morphogen levels, respectively.

Here, we will work only with the nondimensionalized form of equations (2)–(4) (c.f. previous sections). To investigate the variety of patterns produced by the presented model, we have performed more than 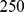 simulations using the nondimensionalized parameters 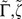 and 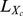 (setting 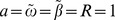, 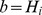 and 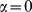, c.f. previous sections). Furthermore, we have considered different values of 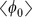. Depending on the exact choice of parameters, simulation results show two different types of long time behavior: either the system tends to equilibrium with a symmetric medium curved pattern and seems to be stable at least for simulated times (c.f. Fig. 2 B), or strong budding appears (c.f. Fig. 2 A) and persists as long as the numerical scheme functions (the adopted numerical discretization is only able to handle medium geometry changes); it is likely that the limiting shape consists of fully budded patches, as experimentally observed e.g. during the formation of new body axes in *Hydra* polyps. For the corresponding simulations we have set 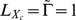 as well as 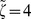 in C but 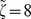 in B. Using different initial conditions (i.e. different values of 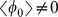 on an undeformed sphere) we find that the equilibrium pattern is independent of the exact choice of initial conditions (c.f. Fig. 2 C) and corresponding equilibrium patterns are very similar (c.f. Fig. 2 D). Even starting with no morphogen (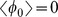) and stochastically perturbed geometry yields the same result (not shown). Hence, the presented mechanism appears to be asymptotically stable and does not require the existence of any prepattern.

**Figure 3:**
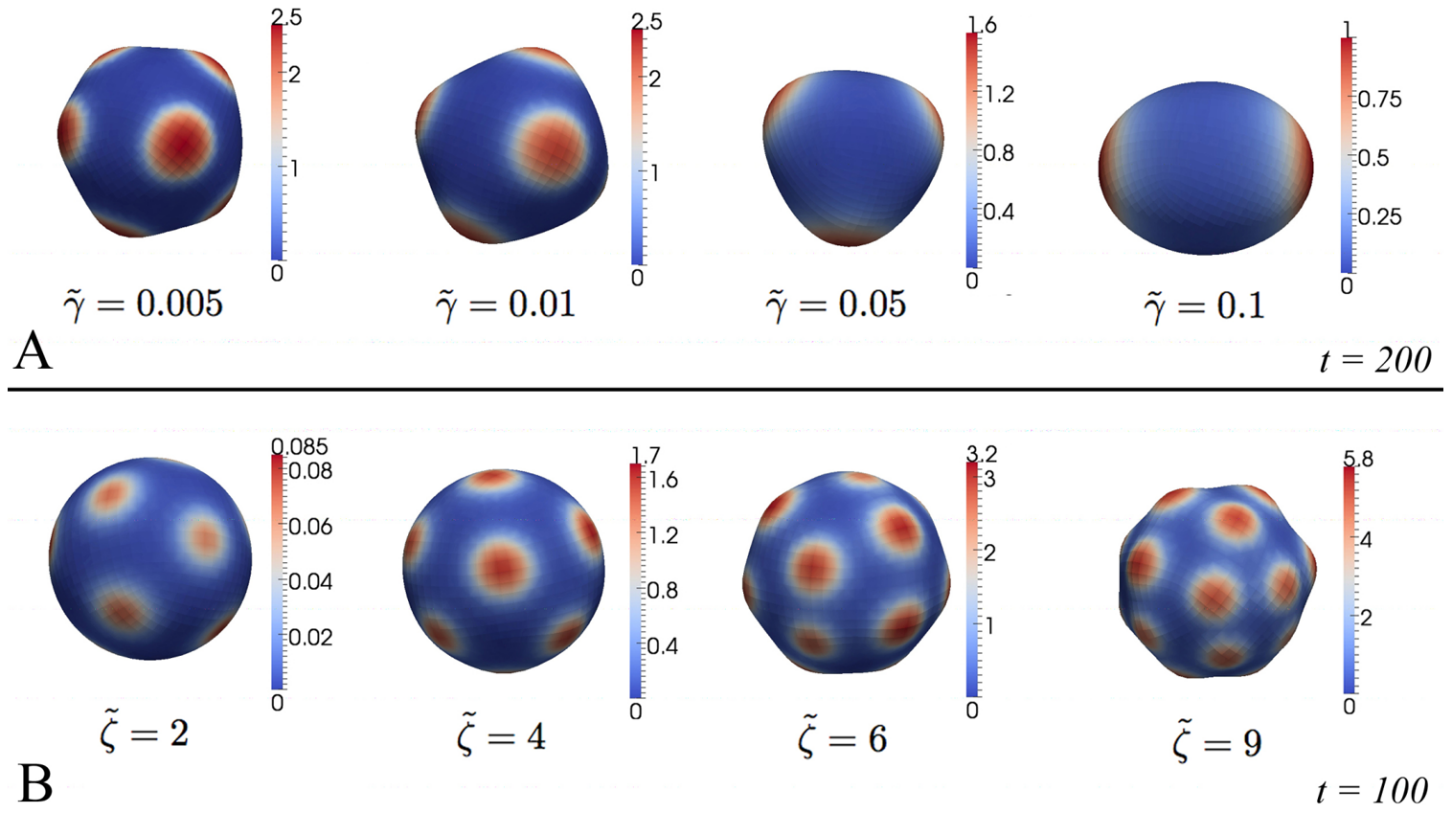
Influence of different model parameters on patterns. A: Increasing diffusion by changing 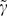 results in bigger morphogen patches. B–C: An increase of coupling between tissue curvature and morphogen expression via 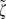 (B) results in more but still equally sized patches. In all simulations red and blue color depict high and low morphogen levels, respectively.

Checking systematically the influence of the varying model parameters 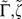 and 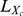 on the corresponding emerging patterns, reveals the following relationships:

- The size of appearing patches can be controlled by diffusion: stepwise increase from 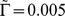 up to 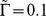 (keeping 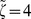 and 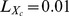 constant) results in larger patches (c.f. Fig. 3 A) and the number of patches decreases down to a minimum of two patches. Higher values of 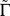 prevent the establishment of patches resulting in a nearly homogeneous sphere (results are not shown).
- The distance between the patches (and hence the number as well, but not the size) and their curvature can be controlled by the strength of coupling between curvature and morphogen expression, i.e. by the curvature dependent production 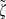: In Fig. 3 B the results of a stepwise increase from 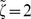 up to 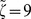 keeping 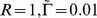 and 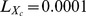 are shown.
- Changing 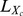 does not result in striking differences in pattern formation but only influences relevant time scales, i.e. the choice of the observed steady state is independent of 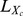. This is expected since possible stationary states are independent of 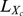 (the whole right hand side of equation (2) is multiplied by 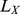). Having in mind that the approach here is based on the assumption of an overdamped motion, 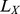 can be interpreted in terms of viscosities. That is, patterns are independent of the specific viscous damping.

Investigating the dynamical behavior of the simulations reveals that all performed simulations typically follow three qualitatively different paths during evolution (c.f. Fig. 2 A–B; for the corresponding simulation we have set 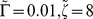, and 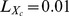.): First, smoothing of initially stochastically distributed morphogen distribution; second, appearance of new and larger slightly curved morphogen patches; and third, distinct curvature patterns coinciding with high morphogen levels, either stabilizing at a symmetric pattern or strongly budding. Interestingly, microscopy data of evolving *Hydra*-reaggregates show similarities to these dynamical patterns [25].

**Figure 4:**
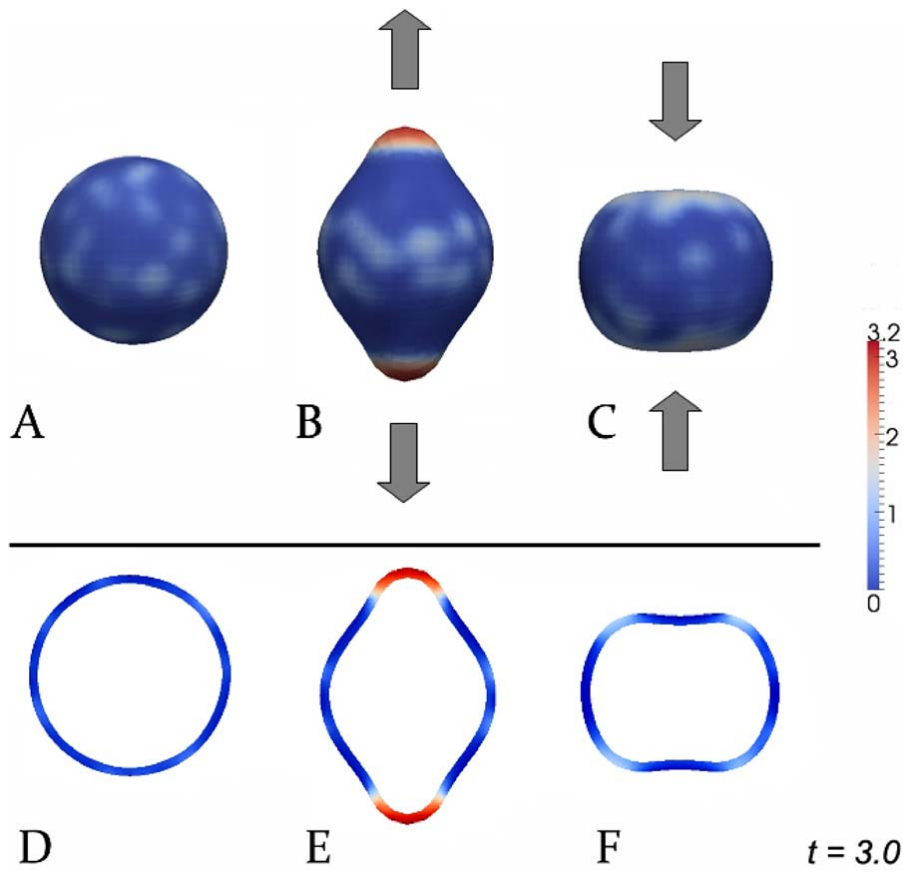
Possible experiments to prove the influence of tissue deformations on morphogen patterns. A–C: 3D-simulations, D–F: corresponding cross-sections. A,D: Wildtype; B,E: pulling outwards; C,F: pressing inwards. In all simulations red and blue color depict high and low morphogen levels, respectively.

Apart form the discussion concerning existence and nature of the morphogens (see Introduction), there exist previous mechanochemical models [26], [27] conceived to explain budding processes in polyps. Also, as it is known, reaction-diffusion systems exhibit similar qualitative behavior, although under differing assumptions. In order to motivate experiments proving (or disproving) the proposed mechanism, we have performed ‘virtual experiments’ shown in Fig. 4. Since it is already known that morphogens are able to influence tissue curvature, we focus in these experiments on the effect that tissue curvature induces morphogen production (depending on the direction of tissue curvature): Forcing the tissue to bend locally outwards (e.g. using micropipettes) activates the morphogen level strikingly (Fig. 4 B) whereas inward bending prevents the establishment of morphogen patches in these regions (Fig. 4 C). However, no applied force yields randomly distributed morphogen patches (Fig. 4 A). In all corresponding simulations we have set 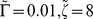, and 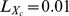 as well as circular pulling and pressing force-terms in B and C, respectively.

To summarize, in this contribution, we have proposed a new non-Turing type model for early pattern formation in tissue development. Based on recent experimental findings, the key assumption is a positive feedback loop between tissue curvature and morphogen production. We have shown numerically that this simple mechanism itself leads to various morphogen and curvature patterns resembling those observed in experiments involving spherical aggregates of *Hydra*-tissue. One of the aims of this paper is to motivate further experimental research to validate the presented mechanism for early pattern formation.

If it is validated by direct experimentation, for example along the lines suggested in Fig. 4, the proposed mechanism will constitute an essential step in the evolution of an initial homogeneous tissue sphere to a complex organism – one of the greatest current mysteries in biology [28], [29].

## Supporting Information

Table S1 General mathematical notations and definitions used in this paper.
(PDF)
