# Supplementary material for: A Mechanochemical Model for Embryonic Pattern Formation: Coupling Tissue Mechanics and Morphogen Expression
Source: PLoS One. 2013 Dec 20;8(12):e82617. doi: 10.1371/journal.pone.0082617 (PMC3869727; doi:10.1371/journal.pone.0082617)
Supplement: Table S1 — General mathematical notations and definitions used in this paper. (PDF) [file pone.0082617.s001.pdf]

A mechanochemical model for embryonic pattern formation: Coupling tissue mechanics and morphogen expression

SUPPORTING INFORMATION

Moritz Mercker, Dirk Hartmann, Anna Marciniak-Czochra

TABLE 1: Notations and definitions

For the convenience of the reader, let us shortly repeat the general notations and definitions used in this paper. For more details we refer to [1].

$\partial_i[.]$	partial derivative with respect to u_i.
$\partial_i\vec{X}$	basis vector of the tangential space, i.e. $\partial_i\vec{X} = \partial_i[\vec{X}]$
$d_t\big[.\big]$	total time derivative,
$(g_{ij})_{i,j}$	first fundamental tensor, $g_{ij} = \partial_i\vec{X} \cdot \partial_j\vec{X}$, where $ds = \sqrt{g}\, d^2u$, and g is its determinant.
$(b_{ij})_{i,j}$	second fundamental tensor, $b_{ij} = -\partial_i\vec{X} \cdot \partial_j\vec{n}$.
g^{ij}	component of the inverse first fundamental tensor,
$\int \ldots ds$	surface integral on a manifold, $ds = \sqrt{g}\, d^2u$.
$\nabla^\Gamma[.]$	first surface gradient: $\nabla^\Gamma\big[f\big] = \sum_{i,j} g^{ij}\partial_j\big[f\big]\partial_i\vec{X}$,
$\Delta^\Gamma\big[.\big]$	first surface Laplacian: $\Delta^\Gamma\big[f\big] = \frac{1}{\sqrt{g}}\sum_{i,j}\partial_i\Big[\sqrt{g}g^{ij}\partial_j[f]\Big]$.
$\delta^\alpha\big[F\big]$	Fréchet-derivative or variation with respect to α,
$\delta F/\delta\vec{X}(\vec{u})$	strong formulation of $\delta^{\vec{X}}\big[F\big]$ in $\vec{X}(\vec{u})$,
H	mean curvature, $H = \mathrm{trace}(\sum_k g^{jk}b_{ij})$,

References

1. Mercker M, Marciniak-Czochra A, Hartmann D (2013) Modeling and computing of deformation dynamics of inhomogeneous biological surfaces. SIAM J Appl Math 73(5): 1768-1792.
